# Ethnic variation in medical and lifestyle risk factors for B cell non-Hodgkin lymphoma: A case-control study among Israelis and Palestinians

**DOI:** 10.1371/journal.pone.0171709

**Published:** 2017-02-14

**Authors:** Geffen Kleinstern, Rania Abu Seir, Riki Perlman, Areej Khatib, Ziad Abdeen, Husein Elyan, Ronit Nirel, Gail Amir, Asad Ramlawi, Fouad Sabatin, Paolo Boffetta, Eldad J. Dann, Meirav Kedmi, Martin Ellis, Arnon Nagler, Dina Ben Yehuda, Ora Paltiel

**Affiliations:** 1 School of Public Health, Hadassah-Hebrew University Medical Organization, Jerusalem, Israel; 2 Dept of Hematology, Hadassah-Hebrew University Medical Center, Jerusalem, Israel; 3 Faculty of Health Professions, Dept of Medical Laboratory Sciences, Al Quds University, Abu Deis, West Bank; 4 Cancer Care Center, Augusta Victoria Hospital, East Jerusalem; 5 School Faculty of Medicine, Dept of Community Medicine, Al Quds University, Abu Deis, West Bank; 6 Beit Jalla Hospital, West Bank, PA; 7 Department of Statistics, Hebrew University, Jerusalem, Israel; 8 Dept of Pathology, Hadassah Medical Center, Jerusalem, Israel; 9 Department of Primary Health Care, Ministry of Health, Ramallah, Palestine; 10 Tisch Cancer Institute and Institute for Translational Epidemiology, Mount Sinai School of Medicine, New York, New York, United States of America; 11 Rambam Medical Center and Rappaport Faculty of Medicine, Technion, Haifa, Israel; 12 Chaim Sheba Medical Center, Tel Hashomer and Tel Aviv University, Israel; 13 Meir Medical Center, Kfar Saba, Israel; Fu Jen Catholic University, TAIWAN

## Abstract

**Background:**

Risk factors for B-cell non-Hodgkin lymphoma (B-NHL) have not been assessed among Palestinian Arabs (PA) and Israeli Jews (IJ).

**Methods:**

In a case-control study we investigated self-reported medical and lifestyle exposures, reporting odds ratios (ORs) and 95% confidence intervals [CIs], by ethnicity, for overall B-NHL and subtypes.

**Results:**

We recruited 823 cases and 808 healthy controls. Among 307 PA/516 IJ B-NHL cases (mean age at diagnosis = 51 [±17] versus 60 [±15] years, respectively) subtype distributions differed, with diffuse large B-cell lymphoma (DLBCL) being prominent among PA (71%) compared to IJ (41%); follicular lymphoma (FL), was observed in 14% versus 28%, and marginal zone lymphoma, in 2% versus 14%, respectively. Overall B-NHL in both populations was associated with recreational sun exposure OR = 1.43 [CI:1.07–1.91], black hair-dye use OR = 1.70 [CI:1.00–2.87], hospitalization for infection OR = 1.68 [CI:1.34–2.11], and first-degree relative with hematopoietic cancer, OR = 1.69 [CI:1.16–2.48]. An inverse association was noted with alcohol use, OR = 0.46 [CI:0.34–0.62]. Subtype-specific exposures included smoking (FL, OR = 1.46 [CI:1.01–2.11]) and >monthly indoor pesticide use (DLBCL, OR = 2.01 [CI:1.35–3.00]). Associations observed for overall B-NHL in PA only included: gardening OR = 1.93 [CI:1.39–2.70]; history of herpes, mononucleosis, rubella, blood transfusion (OR>2.5, P<0.01 for all); while for IJ risk factors included growing fruits and vegetables, OR = 1.87 [CI:1.11–3.15]; and self-reported autoimmune diseases, OR = 1.99 [CI:1.34–2.95].

**Conclusions:**

In these geographically proximate populations we found some unique risk factors for B-NHL. Heterogeneity in the observed associations by ethnicity could reflect differences in lifestyle, medical systems, and reporting patterns, while variations by histology infer specific etiologic factors for lymphoma subtypes.

## Introduction

Non-Hodgkin lymphomas (NHL) are histologically and molecularly heterogeneous malignancies originating from B and T lymphocytes. They account for approximately 3% of the worldwide cancer burden showing global variations in patterns of occurence[1]. NHL incidence rates increased worldwide[2,3] from 1950–2000, tripling in those aged >65[4]. As of 2012, Israel ranked first in the world in NHL incidence rates[5]; NHL represents the fifth most common malignancy in Israel[6], with annual age-adjusted incidence rates of 15.7 and 11.8/10^5^ for Jewish males and females respectively and 10.4 and 10.0/10^5^ respectively for Israeli Arabs[6]. Israel ranks highest in the world in lymphoma incidence according to the World Health Organization, Globocan 2012 (IARC); Lebanon, in close geographic proximity, is ranked second[5]. According to the Palestinian Cancer Registry as of 2014, NHL is the seventh among West Bank Palestinian men and ninth among women[7]. The epidemiology of this important disease has remained relatively unstudied in both these populations.

Several risk factors and exposures have been consistently associated with lymphoma risks, but these explain only a small fraction of NHL risk. Familial aggregation studies[8,9] as well as genetic association studies point to an important hereditary component in the etiology of NHL[10]. Alterations of the immune system by infections[11], autoimmune disorders[12] and immunosuppression[1,13], whether acquired or congenital have been consistently related to NHL susceptibility. The substantially increased incidence rates of NHL observed over the last 50 years strongly suggest the role of environmental factors[13]. These may include rural/agricultural residence, occupational exposures to radiation[3] and certain chemicals (such as insecticides and pesticides), cigarette smoking, and extended use of permanent dark hair dye (all reviewed in Alexander *et al*, 2007). Several exposures have been associated with a decreased risk of NHL including marijuana use[14],alcohol use[3,15,16] and history of blood transfusion[17].

Several risk factors have been found to be subtype-specific[13]. In a large international collaborative analysis including 17,471 NHL cases and 23,096 controls[17], pooled from 20 studies conducted in North America, Europe and Australia [International Lymphoma Epidemiology Consortium (InterLymph)], associations varied among 11 NHL subtypes for medical history factors (autoimmune diseases, hepatitis C virus, eczema, blood transfusion), alcohol consumption, cigarette smoking, and certain occupations. Risks were generally consistent among subtypes for family history of NHL, recreational sun exposure, hay fever, allergies, and socioeconomic status. Autoimmune diseases associated with B-cell activation were more likely to elevate risk for B-cell non-Hodgkin lymphoma (B-NHL). Ethnicity was not explored as a modifying factor in these associations.

In fact, to date, most epidemiological studies of NHL have been carried out in North American and European populations, with a few focusing on East Asian populations. Apart from works from Oman[18] and Egypt[19], few case-control studies have focused on B-NHL in Middle Eastern populations.

Israelis and Palestinians represent genetically and culturally diverse populations living in geographic proximity. Despite sharing the same ecosystem, they differ in terms of lifestyle, health behaviors and medical systems. This is the first large study examining medical history, environmental and lifestyle risk factors for B-NHL and its subtypes in these populations.

## Methods

### Study design

We performed a case-control study which included questionnaires, pathology review, serology and genotyping. This report includes data obtained from questionnaires and histopathology review.

Trained interviewers approached potential cases after confirming potential eligibility of cases (lymphoma type, diagnosis dates etc). Potential controls were approached as detailed below.

### Study population

#### Inclusion criteria

Eligible cases were individuals aged ≥18 years with pathologically confirmed B-NHL recruited from three sources: 1) outpatients and inpatients at Hadassah–Hebrew University Medical Centre, a tertiary center on two campuses, recruited October 2010- March 2014 (N = 507 = 62%); 2) NHL patients from four hospitals in the center and north of Israel (Chaim Sheba, Meir, Rambam and Hadassah Medical Centers) recruited to the Israel Epilymph study in 2003 in (N = 86 = 10%); and 3) outpatients and inpatients attending Nablus, Beit Jalla and Augusta Victoria Hospitals in the West Bank and East Jerusalem (N = 170 = 21%), or recruited using the West Bank Cancer Registry files (N = 60 = 7%), 2009–2013. Efforts were made to recruit cases as soon as possible after diagnosis. The median length of time between diagnosis and interview was three months and 80% of cases were interviewed within 1.5 years of diagnosis.

We obtained, where possible, original pathology reports for all enrolled cases from medical records departments, private laboratories or computerized databases; where these were unavailable we included cases with immunohistochemistry results explicitly recorded in the treating physician’s report. Only cases showing positive immunohistochemistry staining with CD20 or other B-cell markers were included. Cross-referencing of pathology reports was performed with the Israel National Cancer Registry for cases recruited in Israel.

Healthy NHL-free controls were consenting individuals aged ≥18 years frequency-matched to cases by age and sex and recruited from various sources: They included consenting individuals (not including blood relatives) accompanying patients to visits or appointments in 1) hematology clinics and day care at Hadassah–Hebrew University Medical Center for the Israeli Jewish (IJ) cases; and 2) National Hospital of Nablus, Augusta Victoria or Beit Jalla Hospitals and 3) Ministry of Health centers distributed around the West Bank for the Palestinian Arab (PA) cases. Due to occasional consanguineous marriage, spouses were not recruited in the PA population, although they comprised 24% of IJ controls.

**Exclusion criteria** were HIV positivity and inability to provide written informed consent or complete the interview.

### Questionnaire and study variables

Participants were interviewed utilizing a questionnaire adapted from the European Epilymph study[20], and translated into Hebrew, Arabic and Russian. Questionnaire items focused on family history, occupational and chemical exposures, hobbies, history of infection, and immune conditions. Ethnicity for IJ was determined based on place of birth of four grandparents (Ashkenazi, North African, West Asian and Sephardic). For purposes of analysis Arab/Muslim citizens of Israel (N = 5) were analyzed with the PAs. Although participants were not questioned directly about their socio-economic status (SES), education level and frequency of dental visits served as proxies for SES[21].

### Statistical analysis

Differences in the distribution of socio-demographic variables and exposures were assessed using two-sided Fisher’s exact and χ^2^ tests. Demographic variables found to differ in distribution between cases and controls entered as covariates in subsequent models. These included marital status, education (yrs), ethnic origin for IJ and residential region for PA (North, South, Center, other). Conditional logistic regression models were constructed to test associations [reported as odds ratio (OR) and 95% confidence interval (CI)] between B-NHL status and exposures, stratified by population (IJ, PA), frequency matched by sex and age categories (4 year groupings). Individuals with missing data for the exposure variable of interest were excluded from the models. Interactions were assessed between population group (the main effect modifier of interest) and purported risk factor by adding an interaction term into the conditional logistic model. If an interaction was found (interaction P value<0.05) pooling of the populations was not performed. Models were constructed for specific B-NHL subtypes including DLBCL, follicular lymphoma (FL), and MZL (the latter for IJ only, given limited numbers among PA).

Sensitivity analyses were performed to examine the stability of our results for overall B-NHL in the pooled population and subpopulations with the following exclusions:1) Epilymph cases (as they had no concurrent controls); 2) spouse controls; 3) cases diagnosed >1.5 years after recruitment; 4) all three of the above; 5) spouse cases.

Sample size: Based on rates of exposure in controls of ≥5%, two sided α = 0.05, a study with 800 cases and 800 controls provided at least 90% power to detect an OR of 2; a study with 516 cases and 414 controls provided at least 80% power to detect an OR of 2.2; and a study with 307 cases and 294 controls provided at least 80% power to detect an OR of 2.5.

**Fig 1:**
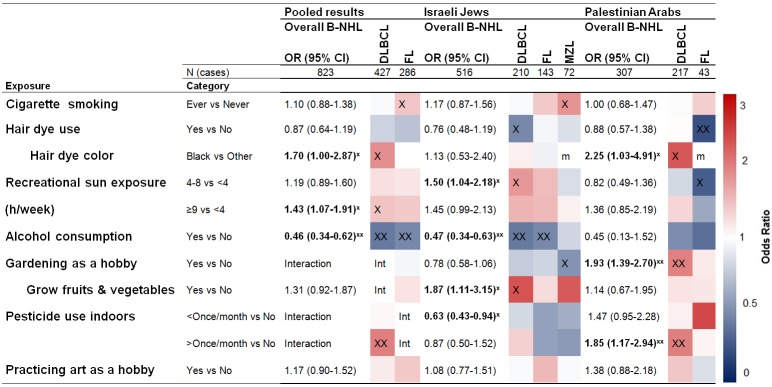
Life style exposures- Adjusted OR for B-NHL and subtypes, overall and by population^□^. Lists the overall odds ratio (OR) with 95% confidence interval (CI) for all risk factors affecting overall B-cell non-Hodgkin lymphoma (B-NHL) and subtypes: diffuse large B-cell lymphoma (DLBCL), follicular lymphoma (FL) and marginal zone lymphoma (MZL), stratified by population (Jews, Arabs), frequency matched by sex and age categories (4 year groupings); adjusted for marital status, education (yrs), ethnic origin for Jews (Ashkenazi, North African, West Asian and Sephardic) and residential region for Arabs (North, South, Center, other). The columns list the exposure category and the OR. The colored grid indicates the OR associated with the exposure for each subtype separately. Red (blue) represents the exposure increases (decreases) risk. ^X^indicates an association with P<0.05, whereas ^XX^indicates P<0.01. *m* indicates missing due to lack of data. *Int* indicates interaction between exposure and sub-populations P<0.05. ^□^based on schema designed by Morton et al.

**Fig 2:**
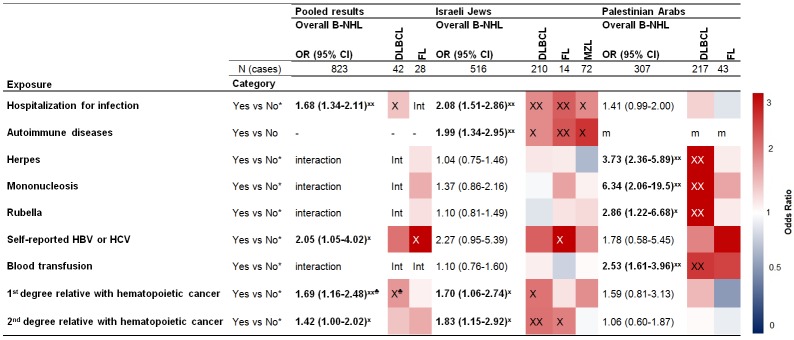
Self-reported medical and family history- Adjusted OR for B-NHL and subtypes, overall and by population^□^. Lists the overall odds ratio (OR) with 95% confidence interval (CI) for all risk factors affecting overall B-cell non-Hodgkin lymphoma (B-NHL) and subtypes: diffuse large B-cell lymphoma (DLBCL), follicular lymphoma (FL) and marginal zone lymphoma (MZL), stratified by population (Jews, Arabs), frequency matched by sex and age categories (4 year groupings); adjusted for marital status, education (yrs), ethnic origin for Jews (Ashkenazi, North African, West Asian and Sephardic) and residential region for Arabs (North, South, Center, other). The columns list the exposure category and the OR. The colored grid indicates the OR associated with the exposure for each subtype separately. Red (blue) represents the exposure increases (decreases) risk. ^X^indicates an association with P<0.05, whereas ^XX^indicates P<0.01. *m* indicates missing due to lack of data. *Int* indicates interaction between exposure and sub-population P<0.05. No* category includes don't know. ^□^based on schema designed by Morton et al. ^♣^Reported by Kleinstern et al.

In general, the alpha level was set at 0.05. We corrected the alpha level for multiple testing using the Bonferroni correction (0.05/157 = 0.000318, based on the number of comparisons performed (shown in Figs 1 and 2), when reporting novel associations or those that were not consistent with previous findings reported by the InterLymph consortium on European/North American populations[17].

### Ethics

The study was approved by the ethics committees of Hadassah, Genetics Committee of the Israel Ministry of Health, and the Institutional Review Board of the Palestinian Ministry of Health. All participants provided written informed consent to participate in this study.

## Results

Response rates were ≥95% for both IJ and PA cases; for controls, response rate was higher for PA (≥95%) compared to IJ (75%). Among recruited PA cases, 77 (20%) were excluded for the following reasons: verified age at diagnosis <18 yrs (N = 23); diagnostic review revealed Hodgkin or T-cell lymphoma (N = 26); inability to confirm B-NHL (N = 28). Among IJ cases, two were excluded due to lack of immune staining (S1 Fig). Of the potentially eligible cases 10% died before recruitment. Thus, our final study population consisted of 823 (307 PA/516 IJ) cases, and 808 healthy controls (394 PA/414 IJ). Demographic characteristics are shown in Table 1. PA participants had substantially larger family sizes than IJ; IJ were more likely to have higher education (>12 years) than PA. Specifically, there are significant differences between PA controls and IJ controls in terms of age, marital status, education level, number of siblings, birth order and frequency of dental visits (P<0.01).

**Table 1:** Demographic characteristics for Israeli Jews and Palestinian Arabs by case-control status for overall B-cell non-Hodgkin lymphoma.

		Israeli Jews						Palestinian Arabs					PA controls vs IJ controls
		Cases Overall		Controls		P*		Cases Overall		Controls		P*	P
Characteristic	Category	N	(%)	N	(%)			N	(%)	N	(%)		
Total No.		516	(100)	414	(100)			307	(100)	394	(100)		
**Sex**	Male	260	(50.4)	185	(44.7)	0.08		154	(50.2)	168	(42.6)	0.05	0.55
	Female	256	(49.6)	229	(55.3)			153	(49.8)	226	(57.4)		
**Age**	<34	33	(6.4)	45	(10.9)	<0.01		64	(20.8)	54	(13.7)	0.05	<0.01
**Years**	35–54	145	(28.1)	111	(26.8)			108	(35.2)	174	(44.2)		
	55–64	135	(26.2)	111	(26.8)			64	(20.8)	85	(21.6)		
	65–74	111	(21.5)	111	(26.8)			47	(15.3)	52	(13.2)		
	≥75	92	(17.8)	36	(8.7)			24	(7.9)	29	(7.3)		
**Marital**	Single	29	(5.6)	19	(4.6)	<0.01		41	(13.4)	34	(8.6)	0.02	<0.01
**Status**	Married	374	(72.6)	382	(92.3)			232	(76.1)	333	(84.5)		
	Other	112	(21.8)	13	(3.1)			32	(10.5)	27	(6.9)		
**Ethnicity/**	Ashkenazi	318	(61.6)	289	(70.0)	<0.01	North	75	(24.7)	78	(19.8)	<0.01	-
**Region**	North African	83	(16.1)	46	(11.1)		Center	120	(39.5)	130	(33.1)		
	West Asian	80	(15.5)	44	(10.7)		South	97	(31.9)	176	(44.8)		
	Sephardic	35	(6.8)	34	(8.2)		Other	12	(3.9)	9	(2.3)		
**Education**	0–8	46	(9.0)	7	(1.7)	<0.01		159	(53.0)	171	(44.3)	0.03	<0.01
**Years**	9–12	154	(30.1)	110	(26.6)			81	(27.0)	108	(28.0)		
	>12	312	(60.9)	297	(71.7)			60	(20.0)	107	(27.7)		
**Number**	0–2	247	(49.2)	227	(54.8)	0.27		21	(6.9)	19	(4.9)	0.50	<0.01
**of**	3–5	157	(31.3)	116	(28.0)			81	(26.5)	101	(25.9)		
**Siblings**	≥6	98	(19.5)	71	(17.2)			203	(66.6)	270	(69.2)		
**Birth order**	1	178	(35.6)	151	(36.5)	0.23		71	(23.4)	69	(18.0)	<0.01	<0.01
	2–3	200	(40.0)	181	(43.7)			115	(37.8)	118	(30.9)		
	≥4	122	(24.4)	82	(19.8)			118	(38.8)	195	(51.1)		
**Frequency of**	≥Once/yr	236	(54.9)	254	(61.5)	0.15		45	(15.0)	61	(16.0)	0.20	<0.01
**dental visits**	Toothache	183	(42.5 (	150	(36.3)			227	(75.4)	298	(78.0)		
	Never	11	(2.6)	9	(2.2)			29	(9.6)	23	(6.0)		

*Comparing cases and controls in this ethnic group.

Values were missing for < 1% of exposure variables.

**Fig 3:**
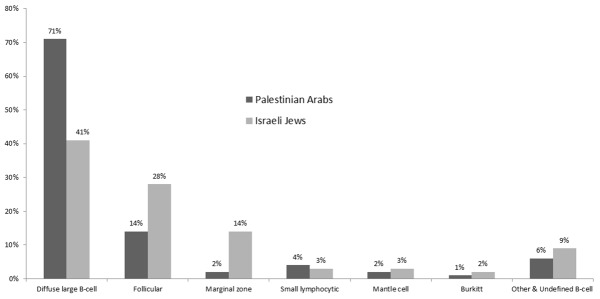
B-cell non-Hodgkin lymphoma (B-NHL) subtype distribution by population.

Histologic distributions differed between populations (Fig 3), with the major subtype being DLBCL in both: (217 (71%) PA vs 210 (41%) IJ). The mean age at diagnosis of B-NHL was 51 years [SD = 17], median 52 years, for PA compared to a mean and median of 60 years [SD = 15] among IJ cases. Extensive demographic characteristics of the study population by subtype are shown in Table A in S1 File. Lifestyle characteristics and environmental exposures are summarized in Table B in S1 File. Table C in S1 File presents medical exposures and family history.

Figs 1 and 2 depict case-control comparisons for the pooled dataset, by population, and by subtype. As shown, certain factors were associated with B-NHL in the overall pooled populations (far left column). These included black hair dye use, recreational sun exposure ≥9h/week versus <4h/week, alcohol use, hospitalization for infection, self-reported hepatitis B or C, and having a first or second degree relative with hematopoietic cancer (the latter was previously reported by Kleinstern *et al*[22]).

In addition, subtype-specific associations were seen in the pooled populations for cigarette smoking (FL) OR = 1.46 (CI:1.01–2.11); black hair dye use (DLBCL) OR = 1.88 (CI:1.03–3.43); recreational sun exposure (DLBCL, P trend<0.0001), ≥9h/week OR = 1.47 (CI:1.03–1.80); and 4-8h/week versus <4h/week OR = 1.25 (CI:0.87–1.80); pesticide use indoors (DLBCL: P trend<0.0001) >once/month OR = 2.01 (CI:1.35–3.00), <once/month OR = 1.07 (CI:0.75–1.52); alcohol use (DLBCL, FL) OR = 0.40 (CI:0.27–0.59), OR = 0.47 (CI:0.30–0.72) respectively; hospitalization for infection (DLBCL) OR = 1.48 (CI:1.12–1.96); having a first degree relative with hematopoietic cancer (DLBCL) OR = 1.83 (CI:1.17–2.87) (previously reported by Kleinstern *et al*[22]).

Regarding population-specific findings, factors that were positively associated with DLBCL in PA only included gardening as a hobby OR = 2.00 (CI:1.38–2.92); history of labial herpes OR = 4.82 (CI:2.90–8.02); mononucleosis OR = 8.42 (CI:2.59–27.3); rubella OR = 3.99 (CI:1.65–9.64); and blood transfusion OR = 2.57 (CI:1.56–4.22).

On the other hand, associations observed exclusively in IJ included fruit and vegetable gardening (DLBCL) OR = 2.35 (CI:1.05–5.26); and a history of auto-immune diseases for DLBCL, OR = 1.91 (CI:1.14–3.21); FL, OR = 2.34 (CI:1.31–4.15); and MZL, OR = 2.60 (CI:1.22–5.52), respectively.

We found significant heterogeneity between PA and IJ in terms of associations with some exposures. Gardening was associated with overall B-NHL and DLBCL only in PA (p = 0.00004, and p = 0.0001, respectively); fruit and vegetable gardening was associated with DLBCL only in IJ; pesticide use indoors >once/month had a positive association with overall B-NHL only in PA. Previous hospitalization for infection was positively associated with FL only in IJ. History of blood transfusion showed a positive association with overall B-NHL, DLBCL and FL only in PA (p = 0.0005, p = 0.002. and p = 0.11, respectively. Strong associations (OR>2) with self-reported herpes, rubella and mononucleosis were observed only among PA. A full description of the observed associations appears in Table D in S1 File.

Sensitivity analyses of the overall B-NHL associations excluding Epilymph cases, spouse controls/cases and prevalent cases did not substantially change the effect sizes or conclusions from the all-inclusive analysis, although statistical precision varied (Table E in S1 File).

## Discussion

In this multicenter case-control study of B-NHL in previously unstudied populations we found that distributions histologic subtypes differed, with DLBCL by far the most common subtype in PA (71%). The histologic distribution for IJ reflects that reported in Western populations[17], for PA the distribution was even more markedly skewed toward DLBCL than that found in Saudi Arabia[23] (51% DLBCL, 7% FL and 5% MZL) or Jordan[24] (62% DLBCL and 14% indolent lymphoma). Jordan’s population is similar genetically and culturally to the PAs. In contrast, a study in Lebanon[25], a country with high NHL incidence[5] reported 44% DLBCL and 20% FL resembling the proportion in our IJ cases.

B-NHL was diagnosed on average nine years younger in PA versus IJ. This most likely due to the younger age structure of the Palestinian population, although it is possible that there is under-diagnosis of cases, especially indolent NHL in older individuals. The median age at diagnosis for IJ is consistent with InterLymph[17]. Here too, findings from Lebanon[25] resembled those in our IJ population, with a mean age at diagnosis of 58 years for DLBCL cases.

Regarding lifestyle factors, both populations exhibited similar associations with NHL. Cigarette smoking was associated with FL only, consistent with Interlymph results[17] as well a cohort study in the United States[26]. Alcohol consumption was protective for all subtypes, although this exposure, as expected, was rarely reported among the mainly Muslim PA. The InterLymph[17] study also reported a negative association between alcohol and DLBCL and MZL. A systematic review including 21 case-control and eight cohort studies reported that alcohol consumption was negatively associated with NHL independent of beverage type[15]. Inhibition of mammalian target of rapamycin (mTOR) signaling in lymphocytes by chronic ethanol exposure[27], has been proposed as a possible mechanism for the favorable role of alcohol on NHL risk.

Recreational sun exposure showed a positive association with DLBCL for the pooled population (but a negative association with FL in PA). This unexpected result may be a chance finding as did not meet the Bonferroni corrected threshold of P<0.00318. It is in contrast to the InterLymph findings[17] which showed a negative association for all B-NHL subtypes. However, a recent US study, while confirming a generally inverse association between sun exposure and lymphoma, found heterogeneity both by subtype and by race/ethnicity[28]. The latitude of Israel/West Bank differs from that in previous studies of NHL and sun exposure, and the general high level of sun exposure in our populations, combined with skin color differences, may explain discrepancies with previously observed relations[29].

Gardening as a hobby was positively associated with overall B-NHL and DLBCL only for PA, and retained its statistical significance even after adjusting for multiple comparisons. This "hobby" was more prevalent in the PA (47.7%) versus IJ population (36.7%), reflecting differences in residential arrangements of the two populations. Most West Bank homes have gardens, used mainly for fruit and olive trees as well as vegetables, and not for flowers or lawns, while the majority of IJ are urbanized and live in apartment buildings. Paradoxically, fruit and vegetable gardening per se, was positively associated with overall B-NHL and DLBCL for IJ only. In both populations these "hobbies" may reflect exposure to pesticides, although other exposures, such as to UV radiation via sun exposure are also possible.

The validity of some self-reported medical history items may be questioned. Despite pre-testing the questionnaire in the PA population, lack of knowledge of medical history and stigma about certain diseases may have limited the accuracy of some responses. This underscores the importance of using biomarkers as unbiased indicators of disease antecedents[22]. The IJ population demonstrated a significant positive association between autoimmune diseases with overall B-NHL and all subtypes consistent with the InterLymph[30] results for DLBCL and MZL whereas responses were blank for 38% of PA participants. History of blood transfusion showed a highly significant positive association for PA only, and the finding persisted after Bonferroni correction. This may be due to more recent hepatitis viral contamination of the blood supply[31,32] or to possible misclassification of exposure during or after diagnosis of B-NHL instead of prior to disease onset. In contrast, InterLymph[17] reported blood transfusion to be protective for B-NHL. Antecedent self-reported viral exposures such as herpes, mononucleosis and rubella were associated with B-NHL in PA only, and there were substantial differences between the populations in reported exposure rates in controls. In addition, hospitalization for infection was positively associated with overall B-NHL and DLBCL in the pooled result, echoing the results in the Jerusalem Perinatal Cohort Study which reported an association between this exposure in infancy and increased risk of aggressive B-NHL[33]. This finding supports the notion that underlying immunodeficiency may be an important risk factor for B-NHL.

As expected, in the pooled results, a positive family history of hematopoietic cancer was positively associated with both overall B-NHL and DLBCL, consistent with InterLymph[8] results, among others, supporting an hereditary component in B-NHL etiology in our region as well.

The strengths of this case-control study include the large sample size in previously unstudied multi-ethnic populations. The high participation rate among cases (≥95%) enhances the representativeness of this sample. A majority of our pooled findings corroborate results in international studies, attesting to the study’s external validity.

Specific challenges in this study include suboptimal recruitment of IJ male controls, as well as potential bias related to the method of control selection. That being said, our controls resembled the general population in terms of ethnic distribution and lifestyle exposures such as smoking[34]. Recruitment strategies based on accompanying friends and visitors has been previously used and validated in cancer epidemiology[35]. Some of the IJ controls were spouses of participating B-NHL cases, and this may have resulted in over-matching for environmental exposures, biasing the study towards the null. However, our analysis strategy, using strata of age and sex, meant that spouse controls were never directly compared to the corresponding case. Furthermore, excluding these controls did not alter the results (Table E in S1 File).

In addition, the possibility of survivorship bias may pertain to the 20% cases recruited >18 months after diagnosis. However, only 10% of the potentially eligible study population died before recruitment reducing the potential for this bias, and sensitivity analysis excluding these cases yielded stable results in the overall B-NHL associations. Finally, the possibility of recall bias and misclassification of exposures ascertained after disease onset (eg blood transfusion), raise the probability of reverse causality. We did not adjust for multiple comparisons; hence some chance findings are possible, although many of the associations had a high prior probability, confirming previously reported findings from the Interlymph pooling projects.

In conclusion, in the Israeli and West Bank Palestinian populations a variety of exposures were related to overall B-NHL. These include infectious, lifestyle and family history exposures. Some were subtype-specific, inferring specific etiologic factors for particular NHL subtypes (eg smoking and FL), which require further investigation as to their mechanisms. Other associations point to substantial etiologic heterogeneity between the populations. Effect modification by ethnicity raises the possibility of gene-environment interactions, but also may reflect differences in diet, cultural habits, socioeconomic, environmental and housing conditions, medical services, exposure to infections in early life or other factors. Cancer epidemiology will be enriched through the broadening of analytic research to include under-studied populations from a variety of ethnicities and geographic regions. This study reflects a unique joint scientific effort involving Israeli and Palestinian investigators, and demonstrates the importance of cooperative research even in politically uncertain climates.

## Supporting information

S1 Fig Flow diagram for recruitment of cases by population. (TIF)

S1 File Supplemental Tables. (PDF)
