# Supplementary figures and images for: Ethnic variation in medical and lifestyle risk factors for B cell non-Hodgkin lymphoma: A case-control study among Israelis and Palestinians
Source: PLoS One. 2017 Feb 14;12(2):e0171709. doi: 10.1371/journal.pone.0171709 (PMC5308607; doi:10.1371/journal.pone.0171709)

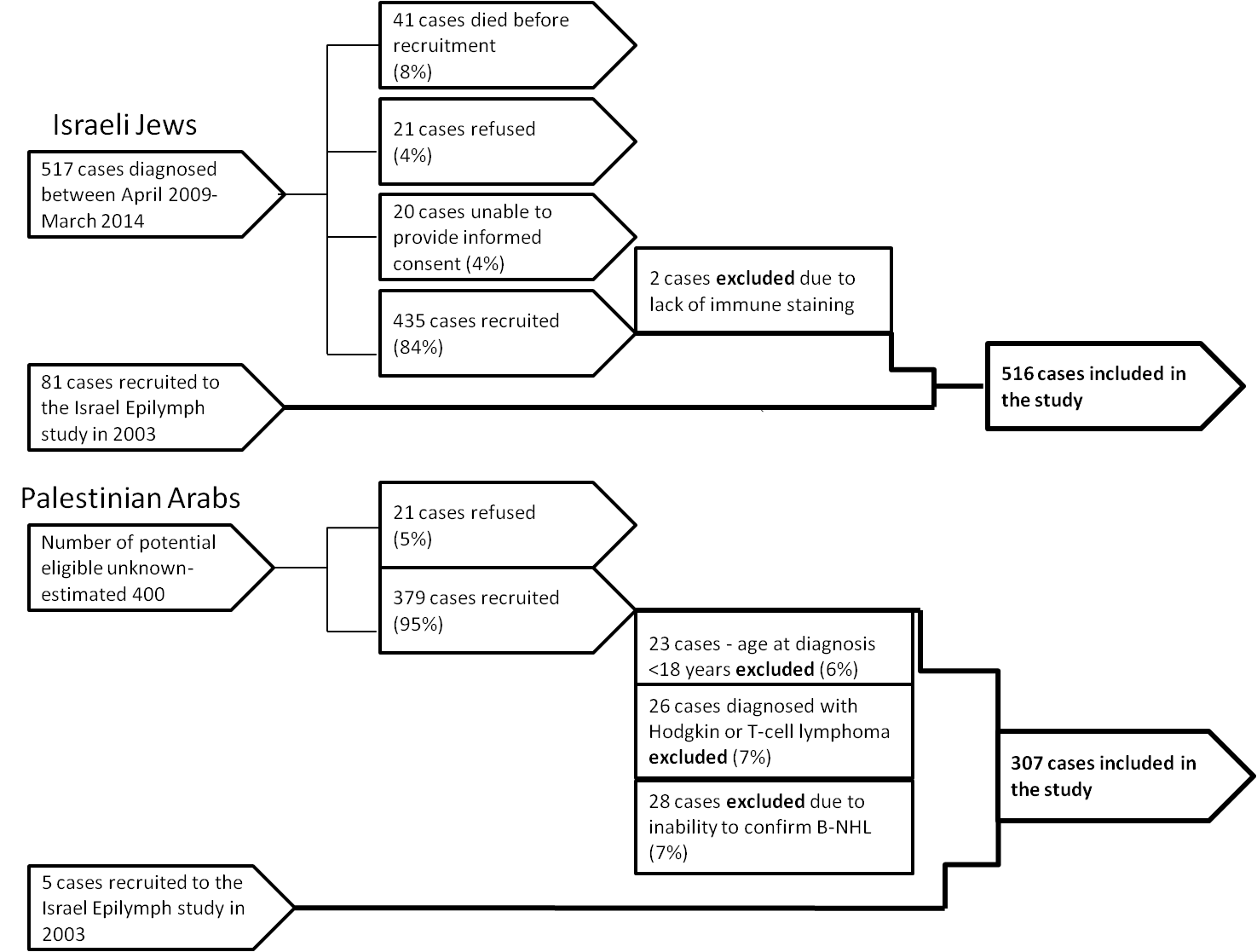

Supplement: S1 Fig — (TIF) [file pone.0171709.s001.tif]
