# Supplementary material for: Ethnic variation in medical and lifestyle risk factors for B cell non-Hodgkin lymphoma: A case-control study among Israelis and Palestinians
Source: PLoS One. 2017 Feb 14;12(2):e0171709. doi: 10.1371/journal.pone.0171709 (PMC5308607; doi:10.1371/journal.pone.0171709)
Supplement: S1 File — (PDF) [file pone.0171709.s002.pdf]

Supplemental Table A: Demographic characteristics for Israeli Jews and Palestinian Arabs by case-control status for overall B-NHL and subtypes

Characteristics		Israeli Jews												Palestinian Arabs								
		DLBCL		FL		MZL		Overall B-NHL		Controls		χ^2 Pvalue*		DLBCL		FL		Overall B-NHL		Controls		χ^2 Pvalue*
		N	(%)	N	(%)	N	(%)	N	(%)	N	(%)		Region	N	(%)	N	(%)	N	(%)	N	(%)	
Total No.		210	(40.7)	143	(27.7)	72	(14.0)	516	(100)	414	(100)			217	(70.7)	43	(14.0)	307	(100)	394	(100)	
Sex	Male	102	(48.6)	69	(48.2)	36	(50.0)	260	(50.4)	185	(44.7)	0.08		99	(45.6)	28	(65.1)	154	(50.2)	168	(42.6)	0.05
	Female	108	(51.4)	74	(51.8)	36	(50.0)	256	(49.6)	229	(55.3)			118	(54.4)	15	(34.9)	153	(49.8)	226	(57.4)	
Age	<34	23	(10.9)	4	(2.8)	2	(2.8)	33	(6.4)	45	(10.9)	<0.01		51	(23.5)	8	(18.6)	64	(20.8)	54	(13.7)	0.05
years	35-54	56	(26.7)	45	(31.5)	25	(34.8)	145	(28.1)	111	(26.8)			76	(35.0)	14	(32.6)	108	(35.2)	174	(44.2)	
	55-64	47	(22.4)	46	(32.1)	17	(23.6)	135	(26.2)	111	(26.8)			36	(16.6)	13	(30.2)	64	(20.8)	85	(21.6)	
	65-74	47	(22.4)	27	(18.9)	14	(19.4)	111	(21.5)	111	(26.8)			35	(16.1)	3	(7.0)	47	(15.3)	52	(13.2)	
	≥75	37	(17.6)	21	(14.7)	14	(19.4)	92	(17.8)	36	(8.7)			19	(8.8)	5	(11.6)	24	(7.9)	29	(7.3)	
Marital	Single	16	(7.6)	5	(3.5)	4	(5.6)	29	(5.6)	19	(4.6)	<0.01		30	(14.0)	5	(11.6)	41	(13.4)	34	(8.6)	0.02
status	Married	146	(69.9)	110	(76.9)	53	(73.6)	374	(72.6)	382	(92.3)			159	(74.0)	38	(88.4)	232	(76.1)	333	(84.5)	
	Other	47	(22.5)	28	(19.6)	15	(20.8)	112	(21.8)	13	(3.1)			26	(12.0)	0	(0.0)	32	(10.5)	27	(6.9)	
Ethnicity/	Ashkenazi	126	(60.0)	90	(62.9)	48	(66.7)	318	(61.6)	289	(70.0)	<0.01	North	57	(26.7)	6	(14.0)	75	(24.7)	78	(19.8)	<0.01
Region	North African	34	(16.2)	29	(20.3)	12	(16.7)	83	(16.1)	46	(11.1)		Center	88	(41.1)	15	(34.9)	120	(39.5)	130	(33.1)	
	West Asian	35	(16.7)	16	(11.2)	9	(12.5)	80	(15.5)	44	(10.7)		South	61	(28.5)	20	(46.5)	97	(31.9)	176	(44.8)	
	Sephardic	15	(7.1)	8	(5.6)	3	(4.1)	35	(6.8)	34	(8.2)		Other	8	(3.7)	2	(4.6)	12	(3.9)	9	(2.3)	
Education	0-8	22	(10.6)	12	(8.4)	4	(5.5)	46	(9.0)	7	(1.7)	<0.01		118	(55.7)	20	(47.6)	159	(53.0)	171	(44.3)	0.03
years	9-12	64	(30.8)	41	(28.9)	21	(29.2)	154	(30.1)	110	(26.6)			52	(24.5)	14	(33.3)	81	(27.0)	108	(28.0)	
	>12	122	(58.6)	89	(62.7)	47	(65.3)	312	(60.9)	297	(71.7)			42	(19.8)	8	(19.1)	60	(20.0)	107	(27.7)	
Number	0-2	97	(46.9)	69	(49.3)	32	(46.4)	247	(49.2)	227	(54.8)	0.27		14	(6.5)	4	(9.3)	21	(6.9)	19	(4.9)	0.50
of	3-5	69	(33.3)	41	(29.3)	25	(36.2)	157	(31.3)	116	(28.0)			61	(28.4)	9	(20.9)	81	(26.5)	101	(25.9)	
siblings	≥6	41	(19.8)	30	(21.4)	12	(17.4)	98	(19.5)	71	(17.2)			140	(65.1)	30	(69.8)	203	(66.6)	270	(69.2)	
Birth order	1	65	(31.4)	54	(38.8)	23	(33.3)	178	(35.6)	151	(36.5)	0.23		47	(22.0)	12	(27.9)	71	(23.4)	69	(18.0)	<0.01
	2-3	89	(43.0)	51	(36.7)	31	(44.9)	200	(40.0)	181	(43.7)			81	(37.8)	16	(37.2)	115	(37.8)	118	(30.9)	
	≥4	53	(25.6)	34	(24.5)	15	(21.8)	122	(24.4)	82	(19.8)			86	(40.2)	15	(34.9)	118	(38.8)	195	(51.1)	
Frequency of	≥Once/yr	98	(54.1)	62	(53.5)	37	(56.9)	236	(54.9)	254	(61.5)	0.15		31	(14.5)	8	(19.0)	45	(15.0)	61	(16.0)	0.20
dental visits	Toothache	78	(43.1)	52	(44.8)	26	(40.0)	183	(42.5)	150	(36.3)			166	(77.6)	27	(64.3)	227	(75.4)	298	(78.0)	
	Never	5	(2.8)	2	(1.7)	2	(3.1)	11	(2.6)	9	(2.2)			17	(7.9)	7	(16.7)	29	(9.6)	23	(6.0)	

B-NHL=B-cell non-Hodgkin lymphoma; DLBCL= diffuse large B-cell lymphoma; FL=follicular lymphoma; MZL=marginal zone lymphoma
*P-value calculated for overall B-NHL versus controls
Values were missing for < 1% for exposure variables

Supplemental Table B: Lifestyle and environmental factors for Israeli Jews and Palestinian Arabs by case-control status for overall B-NHL and subtypes

Characteristics		Israeli Jews										Palestinian Arabs							
		**DLBCL**		**FL**		**MZL**		**Overall B-NHL**		**Controls**		**DLBCL**		**FL**		**Overall B-NHL**		**Controls**	
		**N**	**(%)**	**N**	**(%)**	**N**	**(%)**	**N**	**(%)**	**N**	**(%)**	**N**	**(%)**	**N**	**(%)**	**N**	**(%)**	**N**	**(%)**
Total No.		210	(40.7)	143	(27.7)	72	(14.0)	516	(100)	414	(100)	217	(70.7)	43	(14.0)	307	(100)	394	(100)
Cigarette smoking	Ever	101	(48.8)	84	(60.0)	40	(56.3)	272	(53.7)	196	(47.9)	85	(39.3)	25	(58.1)	128	(41.8)	153	(39.0)
	Never	106	(51.2)	56	(40.0) e	31	(43.7)	235	(46.3)	213	(52.1)	131	(60.7)	18	(41.9)	178	(58.2)	239	(61.0)
Hair dye use	Yes	74	(40.9)	57	(49.1)	28	(43.1)	187	(43.2)	196	(47.3)	85	(39.7)	8	(19.0)	111	(36.9)	174	(44.6)
	No	107	(59.1)	59	(50.9)	37	(56.9)	246	(56.8)	218	(52.7)	129	(60.3)	34	(81.0)	190	(63.1)	216	(55.4)
Used hair dye before 1980	< 1980	24	(32.4)	12	(21.8)	7	(25.9)	50	(27.5)	38	(19.7)	14	(17.5)	0	(0.0)	16	(15.5)	19	(12.6)
	≥ 1980	50	(67.6)	43	(78.2)	20	(74.1)	132	(72.5)	155	(80.3)	66	(82.5)	7	(100)	87	(84.5)	132	(87.4)
Hair dye color	Black	16	(21.6)	7	(12.3)	0	(0.0)	30	(16.2)	23	(11.9)	19	(22.6)	1	(12.5)	26	(23.8)	22	(13.2)
	Other	58	(78.4)	50	(87.7)	28	(100)	155	(83.8)	170	(88.1)	65	(77.4)	7	(87.5)	83	(76.2)	144	(86.8)
Recreational sun exposure (h/week)	<4	41	(22.9)	27	(23.7)	22	(33.8)	104	(24.3)	131	(31.9)	46	(27.1)	10	(31.3)	64	(27.1)	81	(29.0)
	4-8	74	(41.3)	46	(40.3)	20	(30.8)	170	(39.7)	149	(36.2)	48	(28.2)	7	(21.2)	63	(26.7)	93	(33.3)
	≥9	64	(35.8)	41	(36.0)	23	(35.4)	154	(36.0)	131	(31.9)	76	(44.7)	16	(48.5)	109	(46.2)	105	(37.7)
Alcohol consumption	Ever	88	(43.8)	68	(49.6)	40	(56.3)	239	(48.5)	267	(65.0)	4	(4.6)	1	(4.0)	5	(3.9)	8	(4.8)
	Never	113	(56.2)	69	(50.4)	31	(43.6)	254	(51.5)	144	(35.0)	83	(95.4)	24	(96.0)	124	(96.1)	159	(95.2)
Gardening as a hobby	Yes	68	(33.3)	52	(38.0)	17	(23.6)	171	(34.2)	164	(39.7)	117	(54.7)	22	(51.2)	167	(55.1)	163	(42.0)
	No	136	(66.7)	85	(62.0)	55	(76.4)	329	(65.8)	249	(60.3)	97	(45.3)	21	(48.8)	136	(44.9)	225	(58.0)
Grows fruits & vegetables	Yes	40	(66.7)	24	(54.5)	11	(68.8)	92	(61.3)	82	(49.7)	85	(70.8)	16	(72.7)	120	(71.4)	123	(73.2)
	No	20	(33.3)	20	(45.5)	5	(31.2)	58	(38.7)	83	(50.3)	35	(29.2)	6	(27.3)	48	(28.6)	45	(26.8)
Pesticide use indoors	<once/month	33	(18.3)	15	(12.8)	7	(11.1)	65	(15.1)	47	(11.5)	64	(35.4)	5	(14.7)	80	(32.4)	69	(22.0)
	>once/month	120	(66.7)	73	(62.4)	41	(65.1)	274	(63.7)	296	(72.6)	57	(31.5)	17	(50.0)	88	(35.6)	108	(34.4)
	No	27	(15.0)	29	(24.8)	15	(23.8)	91	(21.2)	65	(15.9)	60	(33.1)	12	(35.3)	79	(32.0)	137	(43.6)
Art as a hobby	Yes	49	(24.5)	44	(32.6)	15	(20.8)	124	(25.1)	101	(24.4)	36	(17.0)	5	(11.6)	49	(16.3)	53	(13.8)
	No	151	(75.5)	91	(67.4)	57	(79.2)	370	(74.9)	313	(75.6)	176	(83.0)	38	(88.4)	252	(83.7)	331	(86.2)

B-NHL=B-cell non-Hodgkin lymphoma; DLBCL= diffuse large B-cell lymphoma; FL=follicular lymphoma; MZL=marginal zone lymphoma
Values were missing for < 5% for exposure variables

Supplemental Table C: Medical and family history exposure for Israeli Jews and Palestinian Arabs by case-control status for overall B-NHL and subtypes

Characteristics	Israeli Jews										Palestinian Arabs							
	DLBCL		FL		MZL		Overall B-NHL		Controls		DLBCL		FL		Overall B-NHL		Controls	
	N	(%)	N	(%)	N	(%)	N	(%)	N	(%)	N	(%)	N	(%)	N	(%)	N	(%)
Total No.	210	(40.7)	143	(27.7)	72	(14.0)	516	(100)	414	(100)	217	(70.7)	43	(14.0)	307	(100)	394	(100)
Hospitalization for infection	65	(34.4)	53	(40.8)	26	(38.2)	176	(38.1)	98	(23.7)	73	(34.1)	11	(26.2)	106	(35.1)	115	(29.7)
Herpes	53	(25.7)	39	(29.1)	14	(19.4)	124	(24.9)	98	(23.7)	67	(33.2)	6	(14.6)	87	(30.4)	42	(12.6)
Mononucleosis	20	(9.8)	18	(13.2)	8	(11.3)	58	(11.7)	41	(9.9)	15	(7.4)	1	(2.4)	18	(6.3)	5	(1.5)
Rubella	64	(30.9)	63	(45.6)	30	(42.3)	192	(38.2)	155	(37.4)	21	(10.2)	1	(2.4)	23	(8.0)	9	(2.7)
Autoimmune diseases*	44	(22.1)	27	(20.6)	16	(23.9)	105	(22.3)	57	(13.8)	12	(8.1)	2	(7.7)	15	(7.2)	1	(0.5)
Blood transfusion	36	(18.5)	18	(13.3)	15	(21.1)	89	(18.3)	76	(18.4)	54	(25.1)	9	(20.9)	75	(24.7)	47	(12.2)
1st degree relatives have hematopoietic cancer	28	(14.2)	13	(9.9)	10	(14.3)	62	(13.1)	34	(8.2)	14	(6.5)	2	(4.7)	21	(6.9)	21	(5.4)
2nd degree relatives have hematopoietic cancer	30	(15.8)	21	(16.9)	4	(5.6)	62	(13.7)	35	(8.5)	20	(9.3)	4	(9.3)	28	(9.2)	34	(8.7)

B-NHL=B-cell non-Hodgkin lymphoma; DLBCL= diffuse large B-cell lymphoma; FL=follicular lymphoma; MZL=marginal zone lymphoma
Values were missing for < 5% for exposure variables
*Autoimmune disease was incompletely reported among the Arab participants (38% missing values)

Supplemental Table D: : Life style exposures, self reported medical and family history - Adjusted OR for B-NHL and subtypes, overall and by population

		**Pooled Results**			**Israeli Jews**				**Palestinian Arabs**		
**Characteristics**		**Overall B-NHL OR (95% CI)**	**DLBCL**	**FL**	**Overall B-NHL OR (95% CI)**	**DLBCL**	**FL**	**MZL**	**Overall B-NHL OR (95% CI)**	**DLBCL**	**FL**
	N (cases)	823	427	286	516	210	143	72	307	217	43
Cigarette smoking	Ever vs Never	1.10 (0.88-1.38)	0.98 (0.75-1.30)	**1.46 (1.01-2.11)**[x]	1.17 (0.87-1.56)	0.98 (0.67-1.45)	1.43 (0.93-2.20)	**1.85 (1.02-3.33)**[x]	1.00 (0.68-1.47)	0.99 (0.64-1.53)	1.38 (0.61-3.09)
Hair dye use	Yes vs No	0.87 (0.64-1.19)	0.80 (0.55-1.16)	0.73 (0.41-1.31)	0.76 (0.48-1.19)	**0.47 (0.26-0.85)**[x]	0.96 (0.46-2.00)	0.86 (0.35-2.11)	0.88 (0.57-1.38)	1.03 (0.62-1.70)	**0.22 (0.07-0.68)**[xx]
Hair dye color	Black vs Other	**1.70 (1.00-2.87)**[x]	**1.88 (1.03-3.43)**[x]	1.00 (0.33-3.02)	1.13 (0.53-2.40)	1.13 (0.42-3.05)	0.94 (0.28-3.23)	m	**2.25 (1.03-4.91)**[x]	**2.32 (1.01-5.35)**[x]	**m**
		Pt<.0001 / P=0.049	Pt<.0001 / P=0.10	Pt<.001 / P=0.38	Pt<.0001 / P=0.07	Pt<.0001 / P=0.07	Pt<.0001 / P=0.27	Pt<.001 / P=0.75	Pt<.001 / P=0.08	Pt<.001 / P=0.18	Pt=0.23 / p=0.12
Recreational sun exposure	4-8 vs <4	1.19 (0.89-1.60)	1.25 (0.87-1.80)	1.21 (0.74-1.99)	**1.50 (1.04-2.18)**[x]	**1.81 (1.09-3.02)**[x]	1.53 (0.86-2.73)	0.85 (0.41-1.73)	0.82 (0.49-1.36)	0.83 (0.47-1.46)	**0.28 (0.08-0.96)**[x]
(h/week)	≥9 vs <4	**1.43 (1.07-1.91)**[x]	**1.47 (1.03-1.80)**[x]	1.42 (0.87-2.32)	1.45 (0.99-2.13)	1.59 (0.94-2.70)	1.55 (0.85-2.82)	1.11 (0.55-2.23)	1.36 (0.85-2.19)	1.33 (0.78-2.26)	0.61 (0.23-1.65)
Alcohol consumption	Yes vs No	**0.46 (0.34-0.62)**[xx]	**0.40 (0.27-0.59)**[xx]	**0.47 (0.30-0.72)**[xx]	**0.47 (0.34-0.63)**[xx]	**0.40 (0.26-0.60)**[xx]	**0.47 (0.30-0.75)**[xx]	0.82 (0.45-1.47)	0.45 (0.13-1.52)	0.49 (0.13-1.84)	0.36 (0.04-3.25)
Gardening as a hobby	Yes vs No	interaction	Interaction	0.96 (0.67-1.37)	0.78 (0.58-1.06)	0.77 (0.52-1.16)	0.81 (0.52-1.26)	**0.52 (0.28-0.98)**[x]	**1.93 (1.39-2.70)**[xx]	**2.00 (1.38-2.92)**[xx]	1.17 (0.58-2.41)
Grow fruits & vegetables	Yes vs No	1.31 (0.92-1.87)	Interaction	1.22 (0.66-2.25)	**1.87 (1.11-3.15)**[x]	**2.35 (1.05-5.26)**[x]	1.28 (0.57-2.86)	2.30 (0.70-7.65)	1.14 (0.67-1.95)	1.22 (0.66-2.26)	1.20 (0.38-3.78)
			Pt<.0001 / P=<.001		Pt<.0001 / P=0.04	Pt<.0001 / P=0.21	Pt<.0001 / P=0.16	Pt<.001 / P=0.42	Pt<.0001 / P=0.02	Pt<.0001 / P=0.02	Pt=0.22 / P=0.13
Pesticides use indoors	<Once/month vs No	interaction	1.07 (0.75-1.52)	interaction	**0.63 (0.43-0.94)**[x]	0.83 (0.48-1.45)	0.58 (0.33-1.02)	0.64 (0.31-1.31)	1.47 (0.95-2.28)	1.13 (0.69-1.88)	2.45 (0.98-6.13)
	>Once/month vs No	interaction	**2.01 (1.35-3.00)**[xx]	interaction	0.87 (0.50-1.52)	1.37 (0.66-2.85)	0.58 (0.25-1.32)	0.55 (0.17-1.73)	**1.85 (1.17-2.94)**[xx]	**2.01 (1.20-3.35)**[xx]	1.10 (0.33-3.69)
Practicing art as a hobby	Yes vs No	1.17 (0.90-1.52)	1.16 (0.84-1.60)	1.43 (0.94-2.17)	1.08 (0.77-1.51)	0.98 (0.63-1.54)	1.56 (0.97-2.52)	0.98 (0.48-1.99)	1.38 (0.88-2.18)	1.45 (0.88-2.39)	0.86 (0.30-2.47)
Hospitalization for infection	Yes vs No*	**1.68 (1.34-2.11)**[xx]	**1.48 (1.12-1.96)**[xx]	interaction	**2.08 (1.51-2.86)**[xx]	**1.91 (1.25-2.92)**[xx]	**2.27 (1.43-3.59)**[xx]	**1.91 (1.05-3.48)**[x]	1.41 (0.99-2.00)	1.36 (0.92-2.01)	0.88 (0.40-1.90)
Autoimmune diseases	Yes vs No	m	m	m	**1.99 (1.34-2.95)**[xx]	**1.91 (1.14-3.21)**[x]	**2.34 (1.31-4.15)**[xx]	**2.60 (1.22-5.52)**[x]	**incomplete**	**incomplete**	**incomplete**
Herpes	Yes vs No*	interaction	Interaction	1.23 (0.81-1.87)	1.04 (0.75-1.46)	1.20 (0.78-1.86)	1.16 (0.71-1.88)	0.69 (0.34-1.38)	**3.73 (2.36-5.89)**[xx]	**4.82 (2.90-8.02)**[xx]	1.15 (0.40-3.33)
Mononucleosis	Yes vs No*	interaction	Interaction	1.65 (0.89-3.05)	1.37 (0.86-2.16)	0.97 (0.52-1.80)	1.73 (0.90-3.29)	1.11 (0.46-2.68)	**6.34 (2.06-19.5)**[xx]	**8.42 (2.59-27.3)**[xx]	1.71 (0.16-18.1)
Rubella	Yes vs No*	interaction	interaction	1.25 (0.82-1.89)	1.10 (0.81-1.49)	0.88 (0.59-1.33)	1.23 (0.80-1.90)	1.30 (0.72-2.32)	**2.86 (1.22-6.68)**[x]	**3.99 (1.65-9.64)**[xx]	1.17 (0.12-11.3)
Self reported HBV or HCV	Yes vs No*	**2.05 (1.05-4.02)**[x]	2.11 (0.95-4.67)	**3.33 (1.30-8.57)**[x]	2.27 (0.95-5.39)	2.25 (0.75-6.77)	**3.15 (1.05-9.42)**[x]	1.88 (0.34-10.2)	1.78 (0.58-5.45)	1.98 (0.58-6.76)	2.98 (0.44-20.2)
Blood transfusion	Yes vs No*	Interaction	interaction	interaction	1.10 (0.76-1.60)	1.14 (0.69-1.88)	0.80 (0.44-1.44)	1.04 (0.51-2.12)	**2.53 (1.61-3.96)**[xx]	**2.57 (1.56-4.22)**[xx]	2.49 (0.99-6.21)
1st degree relatives with hematopoietic cancer	Yes vs No*	**1.69 (1.16-2.48)**[xx♣]	**1.83 (1.17-2.87)**[xx♣]	1.12 (0.59-2.13)	**1.70 (1.06-2.74)**[x]	**2.10 (1.16-3.78)**[x]	1.23 (0.59-2.55)	1.89 (0.82-4.40)	1.59 (0.81-3.13)	1.56 (0.72-3.35)	0.55 (0.11-2.64)
2nd degree relatives with hematopoietic cancer	Yes vs No*	**1.42 (1.00-2.02)**[x]	1.47 (0.98-2.21)	1.66 (0.96-2.85)	**1.83 (1.15-2.92)**[x]	**2.12 (1.20-3.72)**[xx]	**2.01 (1.08-3.76)**[x]	0.97 (0.32-2.97)	1.06 (0.60-1.87)	1.04 (0.55-1.97)	0.92 (0.25-3.38)

B-NHL=B-cell non-Hodgkin lymphoma; DLBCL= diffuse large B-cell lymphoma; FL=follicular lymphoma; MZL=marginal zone lymphoma**;** OR=odds ratio; CI=confidence interval;
No* category includes don't know; [X]indicates a statistically significant association P<0.05, whereas [XX]indicates P<0.01; m indicates a large proportion of missing data; Pt=P trend; [♣]Reported by Kleinstern *et al*.

Supplemental Table E: Sensitivity analyses of the overall B-NHL associations (odds ratios), for pooled populations and by population (Israeli Jews and Palestinian Arabs)

		Pooled						Israeli Jews						Palestinian Arabs			
		**Overall B-NHL**	**1**	**2**	**3**	**4**	**5**	**Overall B-NHL**	**1**	**2**	**3**	**4**	**5**	**Overall B-NHL**	**1**	**3**	**6**
	#cases	823	737	823	616	528	724	516	435	516	418	338	417	307	302	195	190
**Characteristics**	# controls	808	808	709	808	709	808	414	414	315	414	315	414	394	394	394	394
Cigarette Smoking	Ever vs Never	1.10	1.08	1.12	0.98	0.97	1.13	1.17	1.09	1.23	1.08	0.02	1.23	1.00	1.02	0.84	0.86
Hair dye use	Yes vs No	0.87	0.87	0.84	0.72	**0.70**^{X}	0.90	0.76	0.76	0.73	**0.58**^{X}	**0.56**^{X}	0.75	0.88	0.88	0.77	0.77
Hair dye color	Black vs Other	**1.70**^{X}	**1.70**^{X}	**1.78**^{X}	**1.86**^{X}	**1.91**^{X}	**1.78**^{X}	1.13	1.13	1.19	1.19	1.22	1.12	**2.25**^{X}	**2.25**^{X}	2.42	2.42
Recreational Sun exposure	4-8 vs <4	1.19	1.19	1.23	**1.46**^{X}	**1.50**^{X}	1.10	**1.50**	**1.50**^{X}	**1.63**^{X}	**2.07**^{XX}	**2.26**^{XX}	1.37	0.82	0.82	0.83	0.83
h/week	≥9 vs <4	**1.43**^{X}	**1.43**^{X}	**1.52**^{XX}	**1.65**^{XX}	**1.76**^{XX}	**1.48**^{X}	1.45	1.45	**1.64**^{X}	**1.90**^{XX}	**2.17**^{XX}	**1.56**^{X}	1.36	1.36	1.34	1.34
Alcohol consumption	Yes vs No	**0.46**^{XX}	**0.50**^{XX}	**0.46**^{XX}	**0.41**^{XX}	**0.43**^{XX}	**0.42**^{XX}	**0.47**^{XX}	**0.51**^{XX}	**0.46**^{XX}	**0.41**^{XX}	**0.45**^{XX}	**0.41**^{XX}	0.45	0.36	0.25	0.12
Gardening as a hobby	Yes vs No	int	-	-	-	-	-	0.78	0.78	0.77	**0.68**^{X}	**0.65**^{X}	0.75	**1.93**^{XX}	**1.94**^{XX}	**1.78**^{XX}	**1.81**^{XX}
Grow fruits & vegetables	Yes vs No	1.31	1.31	1.29	1.23	1.21	1.35	**1.87**^{X}	**1.85**^{X}	**1.82**^{X}	1.64	1.59	**2.16**^{X}	1.14	1.14	1.18	1.18
Pesticides use indoors	<Once/month vs No	int	-	-	-	-	-	**0.63**^{X}	**0.66**^{X}	**0.60**^{X}	**0.63**^{X}	**0.64**^{X}	**0.60**^{X}	1.47	1.47	1.48	1.48
	>Once/month vs No	int	-	-	-	-	-	0.87	0.91	0.78	0.82	0.78	0.79	**1.85**^{XX}	**1.86**^{X}	**1.90**^{X}	**1.90**^{X}
Practicing art as a hobby	Yes vs No	1.17	1.17	1.20	1.20	1.24	1.15	1.08	1.06	1.14	1.10	1.14	1.05	1.38	1.38	1.54	1.54
Hospitalization for infection	Yes vs No*	**1.68**^{XX}	**1.55**^{XX}	**1.64**^{XX}	**1.58**^{XX}	**1.37**^{X}	**1.67**^{XX}	**2.08**^{XX}	**1.78**^{XX}	**2.05**^{XX}	**2.09**^{XX}	**1.72**^{XX}	**2.13**^{XX}	1.41	1.41	1.25	1.25
Autoimmune diseases	Yes vs No	-	-	-	-	-	-	**1.99**^{XX}	**1.92**^{XX}	**2.05**^{XX}	**1.87**^{XX}	**1.83**^{X}	**1.82**^{XX}	m	-	-	-
Herpes	Yes vs No*	int	-	-	-	-	-	1.04	1.14	1.03	1.05	1.14	1.01	**3.73**^{XX}	**3.73**^{XX}	**4.29**^{XX}	**4.03**^{XX}
Mononucleosis	Yes vs No*	int	-	-	-	-	-	1.37	1.47	1.50	1.36	1.62	1.18	**6.34**^{XX}	**6.34**^{XX}	3.38	3.36
Rubella	Yes vs No*	int	-	-	-	-	-	1.10	1.07	1.26	1.18	1.29	1.20	**2.86**^{X}	**2.85**^{X}	**3.88**^{XX}	**3.86**^{XX}
Self reported HBV or HCV	Yes vs No*	**2.05**^{X}	**2.07**^{X}	**2.04**^{X}	**2.04**^{X}	2.06	**2.11**^{X}	2.27	2.27	2.38	**2.53**^{X}	**2.70**^{X}	2.32	1.78	1.78	1.42	1.43
Blood transfusion	Yes vs No*	int	-	-	-	-	-	1.10	1.05	1.15	1.14	1.13	1.13	**2.53**^{XX}	**2.52**^{XX}	**2.04**^{XX}	**2.05**^{XX}
1st degree relatives with hematopoietic cancer	Yes vs No*	**1.69**^{XX}	**1.65**^{X}	**1.58**^{X}	**1.91**^{XX}	**1.76**^{X}	**1.63**^{X}	**1.70**^{X}	**1.63**^{X}	1.56	**1.77**^{X}	1.57	1.57	1.59	1.59	**2.18**^{X}	**2.18**^{X}
2nd degree relatives with hematopoietic cancer	Yes vs No*	**1.42**^{X}	**1.47**^{X}	1.33	**1.54**^{X}	**1.51**^{X}	1.25	**1.83**^{X}	**1.95**^{XX}	**1.67**^{X}	**1.80**^{X}	**1.76**^{X}	1.48	1.06	1.07	1.44	1.47

1:Excluding Epilymph cases; 2:Excluding spouse controls; 3:Excluding cases diagnosed >1.5 years after recruitment ; 4:Excluding 1,2,3; 5: Excluding spouse cases; 6:Excluding 1,3;
No* category includes don't know; Int indicates interaction between exposure and sub-population P<0.05; ^{X}indicates a statistically significant association P<0.05, whereas ^{XX}indicates P<0.01; m indicates a large proportion of missing data; B-NHL=B-cell non-Hodgkin lymphoma.
